# Molecular Dynamics Simulations on the Demolding Process for Nanostructures with Different Aspect Ratios in Injection Molding

**DOI:** 10.3390/mi10100636

**Published:** 2019-09-23

**Authors:** Can Weng, Dongjiao Yang, Mingyong Zhou

**Affiliations:** College of Mechanical and Electrical Engineering, Central South University, Changsha 410083, China; canweng@csu.edu.cn (C.W.); 18198283005@163.com (D.Y.)

**Keywords:** demolding process, molecular dynamics simulation, polypropylene, injection molding, nanostructure, depth-to-width ratio

## Abstract

Injection molding is one of the most potential techniques for fabricating polymeric products in large numbers. The filling process, but also the demolding process, influence the quality of injection-molded nanostructures. In this study, nano-cavities with different depth-to-width ratios (D/W) were built and molecular dynamics simulations on the demolding process were conducted. Conformation change and density distribution were analyzed. Interfacial adhesion was utilized to investigate the interaction mechanism between polypropylene (PP) and nickel mold insert. The results show that the separation would first happen at the shoulder of the nanostructures. Nanostructures and the whole PP layer are both stretched, resulting in a sharp decrease in average density after demolding. The largest increase in the radius of gyration and lowest velocity can be observed in 3:1 nanostructure during the separation. Deformation on nanostructure occurs, but nevertheless the whole structure is still in good shape. The adhesion energy gets higher with the increase of D/W. The demolding force increases quickly to the peak point and then gradually decreases to zero. The majority of the force comes from the adhesion and friction on the nanostructure due to the interfacial interaction.

## 1. Introduction

The functional surface with nanostructures exhibits excellent optical, electrochemical, and biological properties. Typical devices with the functional nanostructured surface, like super-hydrophobic coating, microfluidic chip, and antireflection film [1,2,3,4], are fabricated with the bottom-up methods, such as the self-assembly technology [5], or the top-down techniques, like electron beam lithography [1], ultra-precision milling [6], and nano-molding technology [7]. The fabrication quality of nanostructures plays an important role in its function implementation. Injection molding is one of the most potential techniques for fabricating polymer products in large numbers, with the advantages of cheaper and faster production. It is simple and diversified to fabricate nanostructures on the polymeric surface via injection molding because of the excellent workability, temperature resistance, and high modulus of elasticity [8].

Recently, surface structures with a higher aspect ratio or smaller dimension are being fabricated by injection molding [1,7,9,10]. During the injection molding process, the small dimension and high precision requirements would bring significant challenges, especially when the feature size is down to tens of nanometer or the aspect ratio is relatively high. The marked difference in thermal expansion coefficient between the metal insert and polymer material results in different shrinkages after demolding. The replication quality of nanostructures is quite sensitive to the change of processing parameters, which is not only determined by the filling process, but also the demolding process, due to the scale effect. Common demolding defects, including bending, necking, and structure fracture, would affect the mechanical properties. Moreover, non-destructive demolding is quite essential for achieving the performance of micro/nano-structured surfaces [11,12]. Therefore, it is necessary to analyze the interface behaviors at the nanoscale when the polymer is separated from the metallic mold insert.

The demolding force is mainly composed of adhesion, friction force, and shrinkage that are caused by different thermal expansion coefficients [12,13]. When the processing parameters were optimized to minimize the demolding force, it was found that the adhesion force had a higher influence than the friction force [14]. When considering the scale effect, the interaction mechanism between the polymer and mold insert at the nanoscale still requires further investigation. Experimental researches showed that, when the surface structure/roughness was down to several tens of nanometers, the strength of interfacial interaction, such as van der Waals force, was drastically increased, which forms a strong adhesion between polymer and metal insert [15]. Masato et al. [16] studied the influence of surface roughness of mold insert on the demolding force. If the surface roughness is less than 0.5 μm, the adhesion between the polymer and the insert dominates the final quality during separation.

Simulation methods that are based on continuum mechanics commonly fail to accurately predict the polymer behaviors at the nanoscale, because of the drastic changes in material properties. Recently, molecular dynamics (MD) simulation that is based on the atomistic movement theory provides the potential to study the interface behaviors at the atomic level [17,18]. The MD method has been successfully applied to the study the nano-imprinting [19,20,21,22] and injection molding process [23,24,25]. Even though some researchers are focusing on the demolding process in nano-imprinting, which could give inspiration and guidance to understand the demolding mechanism of polymeric nanostructures in nano-molding process, little literature on the demolding process of nanostructures in injection molding by the MD method is reported up to now. Despite the gaps in time-scale and size-scale between MD method and experimental research, the MD simulation can not only provide insight view of the movement behavior of polymer molecule, but also qualitatively estimate the morphology development in the nanoscale.

In this study, MD simulation models were constructed for the demolding investigation. Nano-cavities with different depth-to-width ratios (D/W) were built. Conformation changes, including molecular behaviors during the ejection, radius of gyration, and demolding velocity, were studied. Meanwhile, the density for both nanostructure and the whole polymer layer were compared to analyze the influence of the D/W. Adhesion energy and demolding force were proposed to understand the inner mechanism of the interfacial interaction between polymer and mold insert during the demolding process.

## 2. Materials and Methods

### 2.1. Materials and Model Constructing

**Figure 1:**
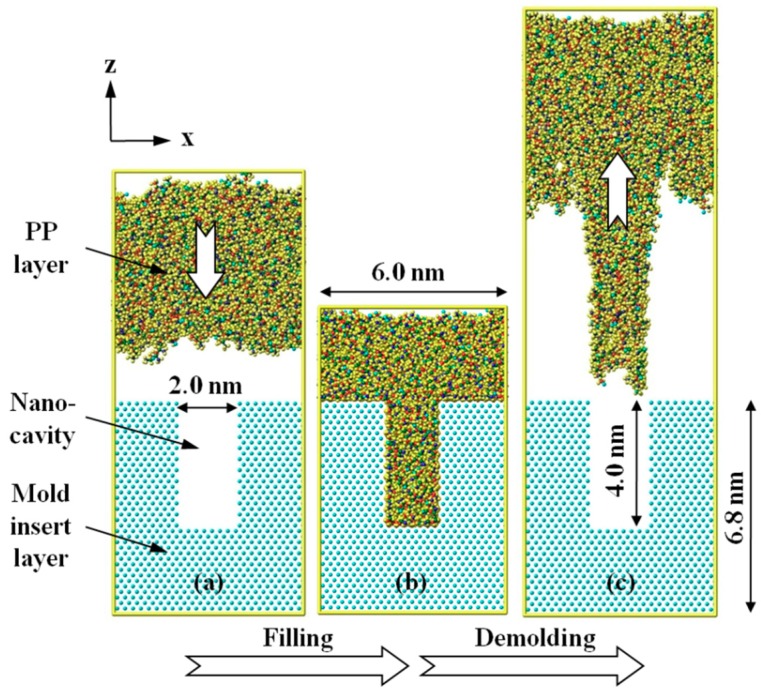
Atomistic model and procedure for the simulation. (**a**) The PP molecules fill the nano-cavity via injection molding, forming the nanostructure after cooling process in order to build (**b**) the initial model for demolding simulation. Afterwards, (**c**) the nanostructure is gradually pulled out from the mold insert.

The simulation system consisted of a polymer layer as the upper layer and a mold insert as the lower layer, as shown in Figure 1. For polymer layer, polypropylene (PP) was selected as the polymer material in nano-injection molding. An atomistic model of PP layer was constructed in a square box with dimensions of 6.0 × 6.0 × 5.3 nm^3^. The initial density of the layer was set to be 0.9 g/cm^3^ at 293 K. There were a total of 200 chains in the box, and the degree of polymerization of each chain was 10. With such a low molecular weight, the nano-cavity can be better filled during the injection molding process, which would be helpful to build an initial model for the demolding simulation. Energy minimization and subsequently anneal treatment were utilized to optimize the conformation of the molecule structure in the PP layer. The layer was then heated up to 523 K to obtain a melt state, according to the actual condition in injection molding.

The mold insert layer was composed of nickel atoms as a FCC structure with (1 0 0) plane. The insert layer had the same dimension in length and width as the PP layer. It is known that the mold insert has a much higher stiffness than the polymer. The insert layer was treated as a rigid body, so that all of the nickel atoms were constrained during the whole simulation. Rectangle nano-cavity was built in the upper of the nickel layer. The width of nano-cavity was set as a constant value of 2.0 nm, while the depths were 2.1 nm, 4.0 nm, and 6.1 nm, respectively. Three insert layers with different D/W were constructed, approximately from 1:1 to 3:1. Period boundary conditions in the x (length) and y (width) directions were used, while non-periodic and shrink-wrapped boundary condition in z (height) direction was set. Subsequently, the PP molecules can be shrinked or stretched during the simulation. The interaction between molecules in the upper region and the nickel atoms could be avoided by such a boundary condition.

### 2.2. Force Field and Simulation Procedure

Polymer Consistent Force Field (PCFF) was adopted to describe the intermolecular and non-bonded interactions between the atoms in the polymer layer. The force field PCFF was based on force field CFF91 with additional parameters being specified for polymer material. It consisted of not only the cross-term potentials, valence potentials, such as bond stretching, angular bending, and torsion potential, but also non-bonded interactions, including Lennard-Jones (9–6) and Coulomb potentials. The non-bonded interaction between PP molecules and nickel atoms was described with Lennard-Jones potential, with a cutoff distance of 1.25 nm.

Sufficient packing during the injection molding process is required in order to construct a demolding model with good replication quality in nano-cavity [26,27]. Hereby, a negative force (*f*_1_) of 1.0 Kcal/mol·Ȧ in the z-direction was applied to the PP layer. The insert layer was kept at a constant mold temperature of 373 K. As a result, the PP layer would gradually cool down to the mold temperature during the filling. The filling process was undertaken within a total of 4.0 ps in a constant particle number, volume, and temperature (NVT) ensemble, with a time step of 0.1 fs. The whole system was further cooled down from 373 K to 353 K in 1.0 ps. The final simulation result was treated as the initial model for the demolding process investigation. Afterwards, an external force (*f*_2_) of 1.0 Kcal/mol·Ȧ along *z*-axis was then applied to the PP layer to release the nanostructure from the insert layer in anotherr NVT ensemble. The demolding process was simulated in a varied time that depended on the D/W of the nano-cavity until the nanostructure was completely pulled out. All of the simulations mentioned above were performed by the Large-scale Atomic/Molecular Massively Parallel Simulator (LAMMPS) [28], which is an open source molecular dynamics package in a computer cluster.

## 3. Result and Discussion

### 3.1. Investigation on the Conformation Change

**Figure 2:**
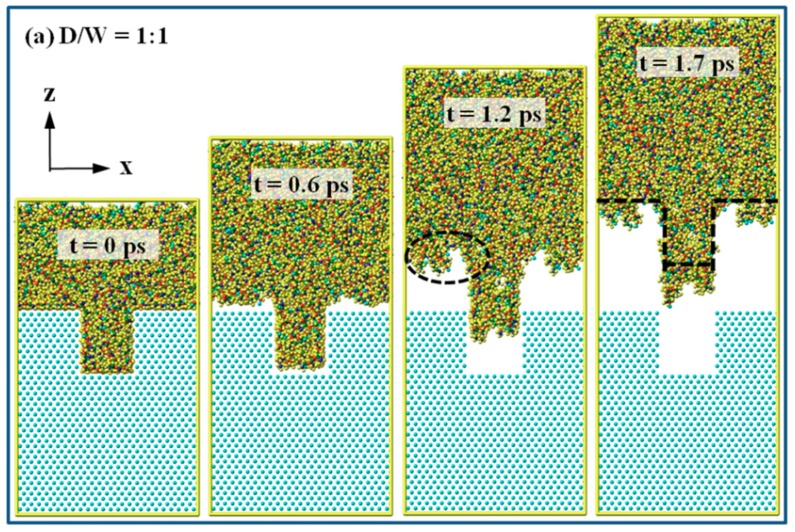

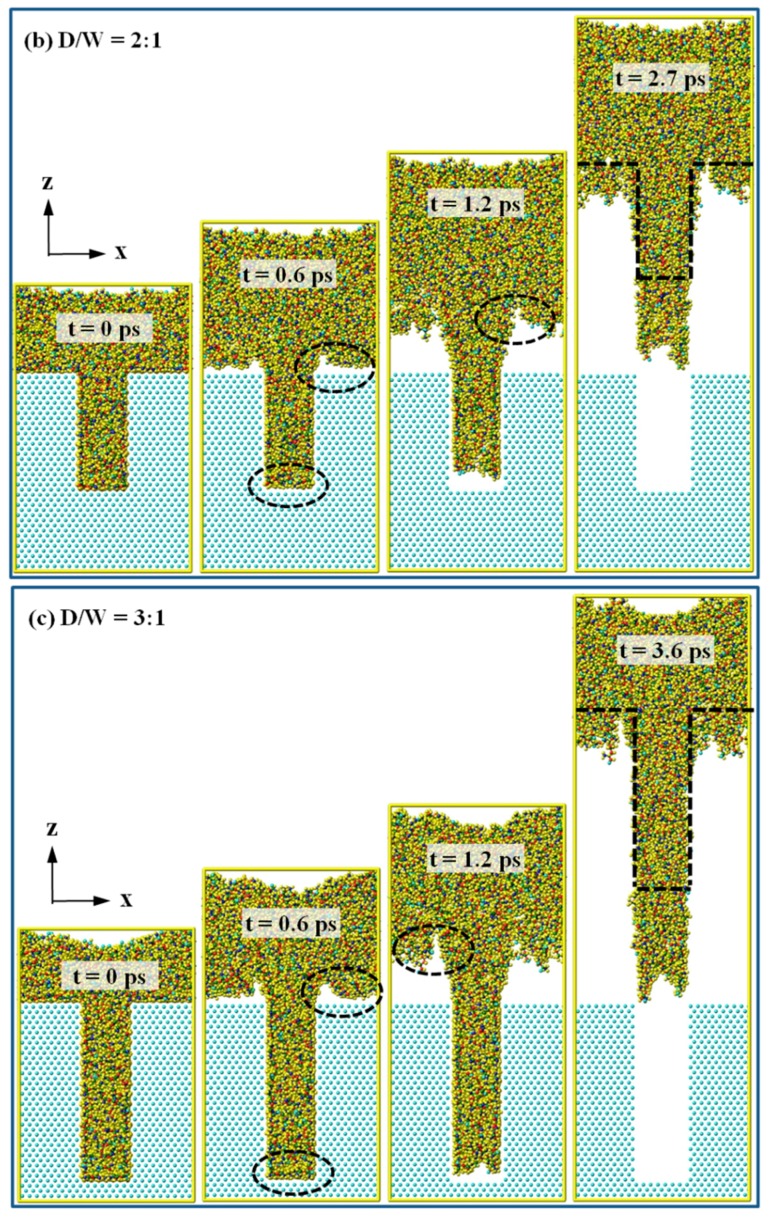
Snapshots of the PP nanostructure during the demolding process, with the D/W of these nano-cavities of (**a**) 1:1, (**b**) 2:1, and (**c**) 3:1, respectively.

Figure 2 shows the snapshots of PP nanostructures with different D/W during the demolding process. The nano-cavity is fully filled during the injection molding process, as shown at 0 ps. It can be seen that there is hardly any separation in 1:1 nano-cavity at the early stage of the demolding process, while the upper molecules begin to show an upward movement. With the process goes on, molecules that are close to the nickel surface tend to fall behind because of the interaction with nickel atoms, as shown at 1.2 ps. Once the nanostructure was pulled out from the nano-cavity, boundary restriction from the insert layer no longer exists. Deformation in nanostructure gradually occurs and a slight expansion at the shoulder is eventually observed. This is mainly because of the interfacial interaction at the shoulder that was formed between PP and nickel atoms during the molding process. Some atoms in PP nanostructure are adhered to the cavity surface. Meanwhile, negative force is generated in molecules that are within the cutoff distance of non-bonded interaction, which thus results in surface unevenness and an elongation of the nanostructure. The nanostructure with D/W of 1:1 is completely released from the mold insert layer at a simulation time of 1.7 ps. With the increase of D/W, the demolding process becomes slower. Nanostructures are stretched during the pull-out process, regardless of the value of the D/W. It indicates that, in order to realize perfect replication fidelity of nanostructure, an anti-sticking treatment is suggested to be done before the demolding process in actual condition. For 2:1 and 3:1 nano-cavities, the separation first happens at the shoulder of the PP nanostructures, as shown at the simulation time of 0.6 ps in Figure 2b,c. Non-bonded interaction strength generated in the injection molding process is shown to be higher because there are more molecules being filled in the nano-cavity with the aspect ratio of 2:1 and 3:1. For 2:1 and 3:1 nano-cavities, the corresponding times for the complete separation are 2.7 ps and 3.6 ps, respectively. Although deformation in nanostructure can be observed during the ejection process, the integrity of each nanostructure is still in good shape generally.

**Figure 3:**
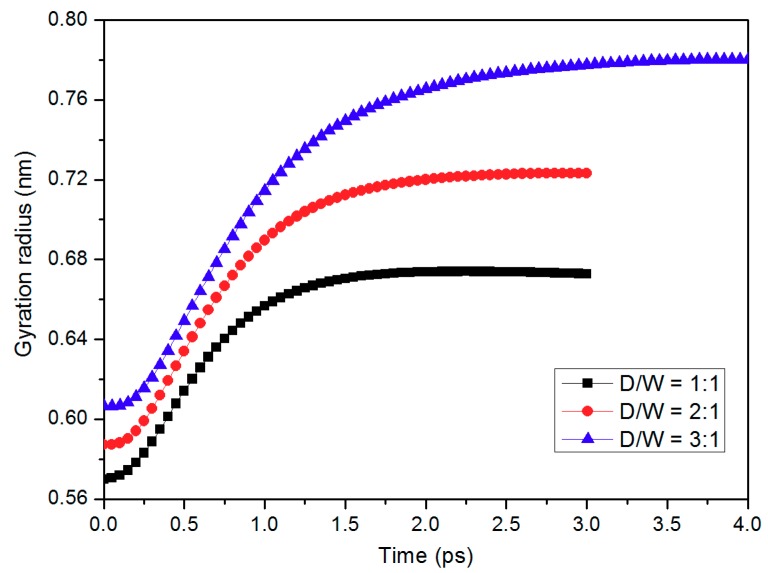
Gyration radii of polypropylene (PP) chain during the demolding process with different depth-to-width ratios (D/W).

The radius of gyration is commonly used as an indicator for the molecular size of polymer chains [29]. In this study, the mean square radius of gyration was introduced to analyze the conformation change of PP chains during the demolding process. It is demonstrated in Figure 3 that the radius first increases and then gradually seems to be equilibrated. It means that these chains are stretched away from the mold insert until the PP layer is completely released. Since more molecule chains are twisted in nano-cavity with 3:1 D/W, it implies greater flexibility for these chains. As a result, the largest change in the radius of gyration can be observed in this case. The radius of gyration reaches to 0.78 nm at the end of the demolding stage, being increased by 28.3%. Additionally, the increases in the radius of gyration are 23.1% for 2:1 nanostructure and 18.1% for 1:1 nanostructure, respectively. In addition, more simulation time is required to reach the equilibrium state when the depth of the nano-cavity is higher. Under combined effects of non-bonded interaction and the demolding force that was applied to the whole layer, the PP chains are greatly stretched, which makes good agreement with the conformation change in Figure 2.

**Figure 4:**
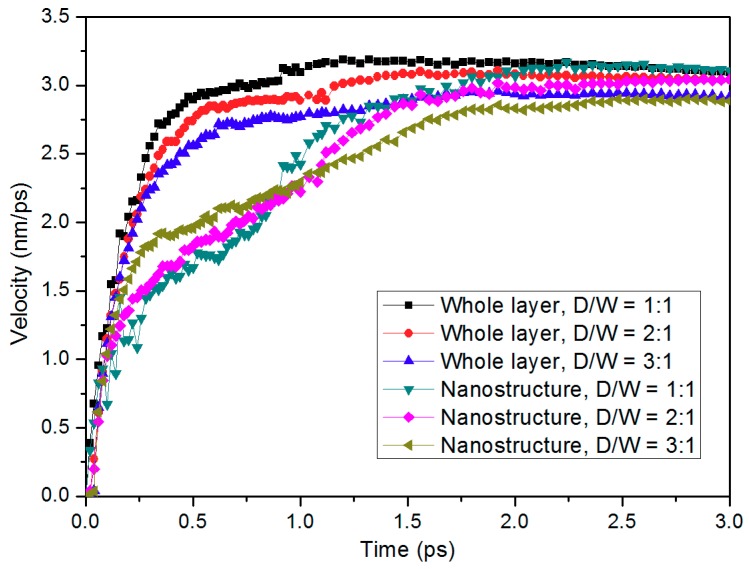
Velocities of the whole PP layer and the PP nanostructure during the demolding process with different D/W.

Velocities in PP layer during the demolding process were calculated in order to investigate the elongation phenomenon of the injection-molded nanostructure. Figure 4 shows the profiles for the average velocities in both nanostructure and the whole layer. The velocity of the whole layer grows fast at the early stage and then reaches to a steady state eventually, approximately 2.9 nm/ps to 3.1 nm/ps. The velocity of PP nanostructure is lower than the whole layer since the PP molecules in nano-cavity are restrained by the interaction with nickel atoms. The velocity difference between nanostructure and the whole layer contributes to elongation growth in nanostructure’s length. PP layer with higher D/W also shows a lower velocity during the demolding process because of the interfacial adhesion. By comparing the velocities of nanostructures with different D/W, it can be found that atoms in the 3:1 nanostructure move faster at the beginning of demolding. This is mainly because the adhesion energy that is generated by the non-bonded interaction is less than triple. Nevertheless, due to the spring back of PP molecules and the decrease of demolding force, the velocity grows fast with the separation of nanostructure in 1:1 nano-cavity. Section 3.3 will discuss further details regarding the interaction energy and demolding strength.

### 3.2. Determination of Density and Its Distribution

**Figure 5:**
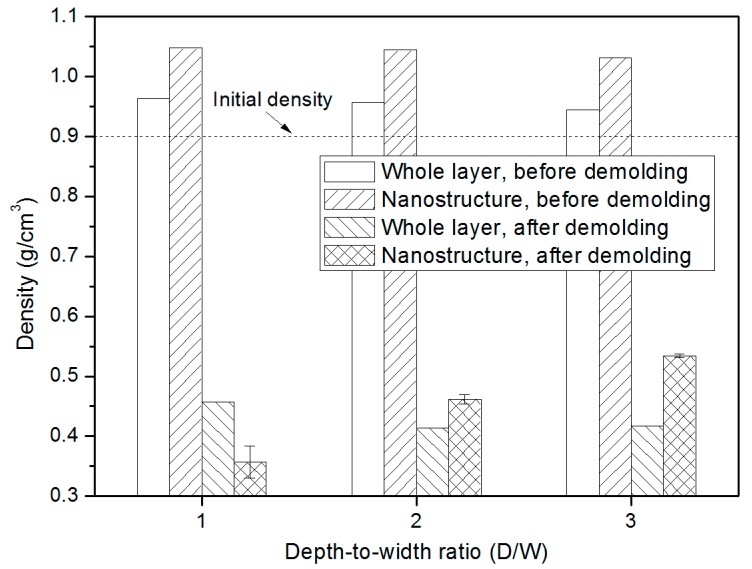
Densities of the whole PP layer and the PP nanostructure with different D/W, compared with the density values before and after demolding.

Density in nanostructure is calculated with the same height as the initial value before the demolding since the outline of PP nanostructure at the end of the demolding process is not so clear. It is demonstrated that both densities of nanostructures and the whole layer before the demolding were higher than the initial density (0.9 g/cm^3^) due to the high pressure that was applied to the PP layer in injection molding. Polymer molecules are forced towards the insert layer, and the nano-cavity is highly filled. Similar results in the density change of the injection molded nanostructure were observed in our previous research [27]. The density of nanostructure remains almost the same, regardless of the aspect ratio. However, the average density dramatically drops when the nanostructure is completely released from the nano-cavity, less than half of the density before demolding, as shown in Figure 5. By analyzing the conformation changes in the radius of gyration and the snapshots during the demolding process, it can be found that the molecule chains in the whole PP layer were stretched. The total heights of both nanostructure and the PP layer are increased, while the corresponding mass almost remains the same.

**Figure 6:**
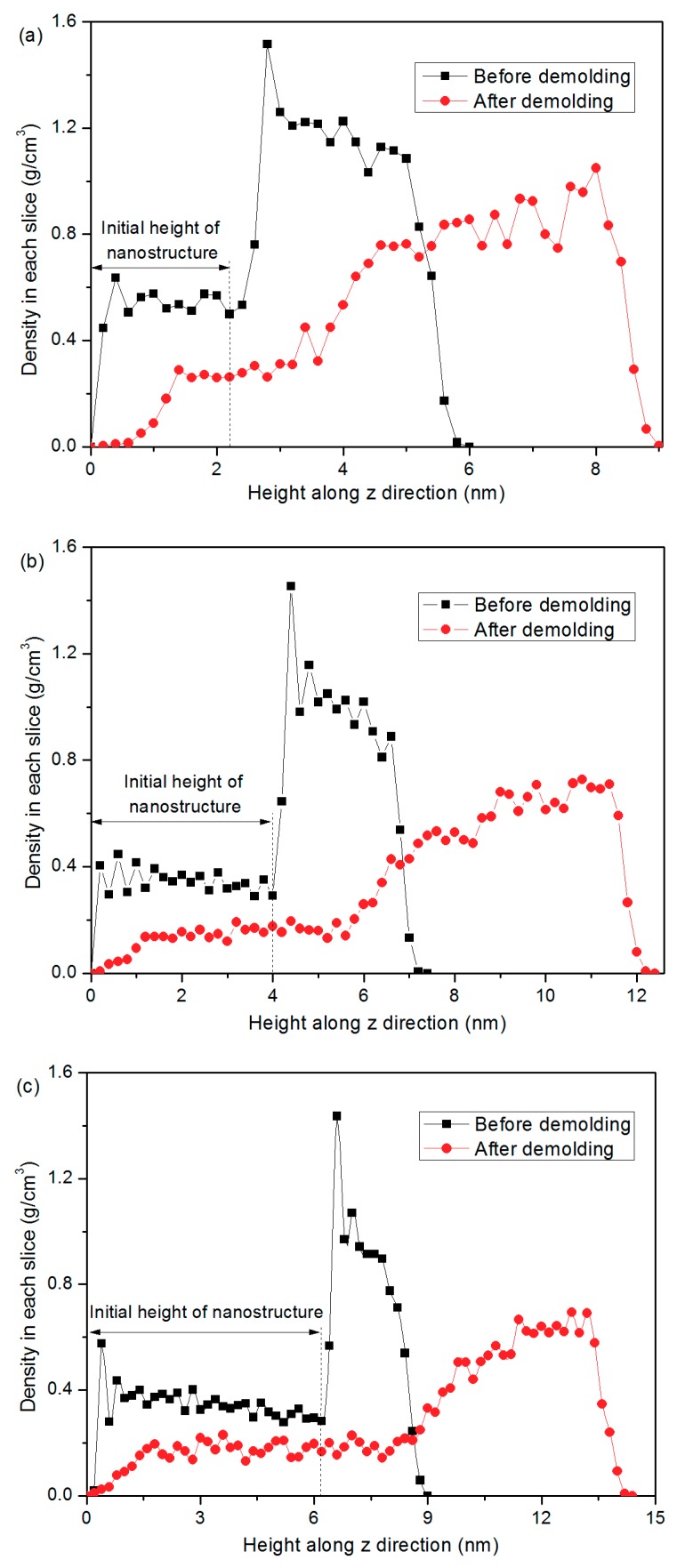
Density profiles in each slice of PP layer before and after the demolding process, with the D/W of these nano-cavities of (**a**) 1:1, (**b**) 2:1, and (**c**) 3:1, respectively.

Figure 6 shows the density distribution of PP molecules in each slice along the *z*-axis, with a slice thickness of 0.2 nm and width of 6.0 nm. The density profile reaches a peak point at the top surface of the insert layer. It means that the PP molecules are enriched at the interface due to the packing pressure and the interfacial interaction between PP and nickel atoms. The interaction contributes to the adhesion force that prevents the PP molecules from separation. The density distribution at the interface further illustrates the reason for the deformation of the shoulder of PP nanostructure. The calculated slice density in PP nanostructure is much lower than the actual density because the width of the nanostructure is 2.0 nm while the width of the whole system is 6.0 nm, as shown in Figure 5. By comparing the density profiles before and after demolding, it can be observed that the total height of the nanostructure that is pulled out after demolding is obviously higher than the initial height, which means that the nanostructure is stretched after the demolding process. The density at the bottom of PP nanostructure shows the lowest value, while the density in the middle area of the nanostructure is relatively stable.

### 3.3. Determination of Adhesion Energy and Demolding Force

**Figure 7:**
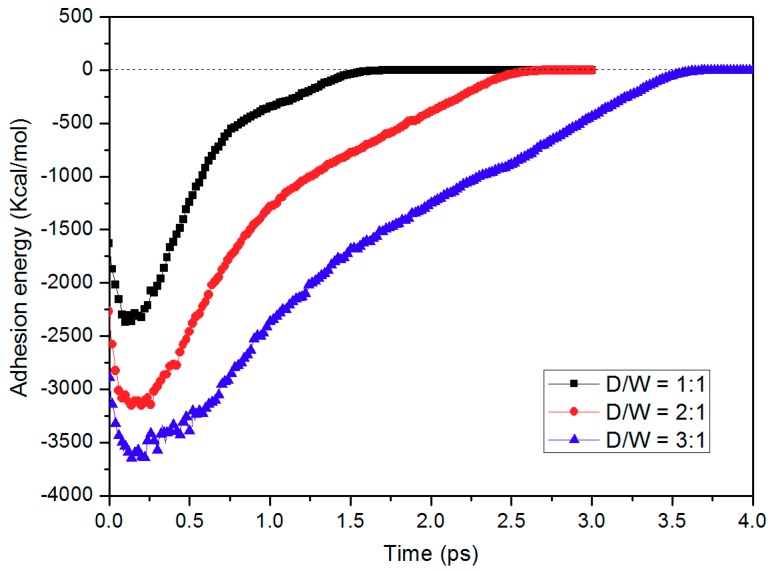
Adhesion energies during the demolding process with different D/W.

According to the previous studies, the adhesion energy between the PP layer and nickel layer is generated by the non-bonded interaction at the interface [30,31,32]. In this study, the adhesion energy could be calculated from Equation (1)

(1) $$E_{adhesion}=E_{interaction}=E_{total}-(E_{PP}+E_{Ni})$$

where *E_total_* is the total potential energy of the whole simulation system, *E_PP_* is the potential energy of PP layer without any contribution from the mold insert, and *E_Ni_* is the potential energy of the nickel layer without PP. The interaction energy is mainly determined by the close contacts between PP and nickel atoms that are within the cut-off distance (1.25 nm) of the non-bonded interaction. Figure 7 shows the adhesion energy distributions with different D/W during the demolding process. The negative value indicates that the PP and nickel atom attract to each other. The absolute value of the adhesion energy rapidly increases at the beginning, which is because the stable state of the initial conformation turns out to be destabilized due to the external releasing force *f*_2_. The adhesion energy decreases shortly afterwards since the nanostructure is gradually pulled out from the nano-cavity. The effective contact area between PP and nickel atom is getting smaller during the demolding process. Maximum adhesion energy is found in the simulation system, with the aspect ratio of 3:1. The highest adhesion energy for 3:1 nano-cavity system is −3645.54 Kcal/mol, while the highest energies for 2:1 and 1:1 system are −3154.52 Kcal/mol and −2364.53 Kcal/mol, respectively. When the PP nanostructure is completely separated from the nano-cavity, the adhesion energy turns to zero, which means that the non-bonded interaction no longer exists.

**Figure 8:**
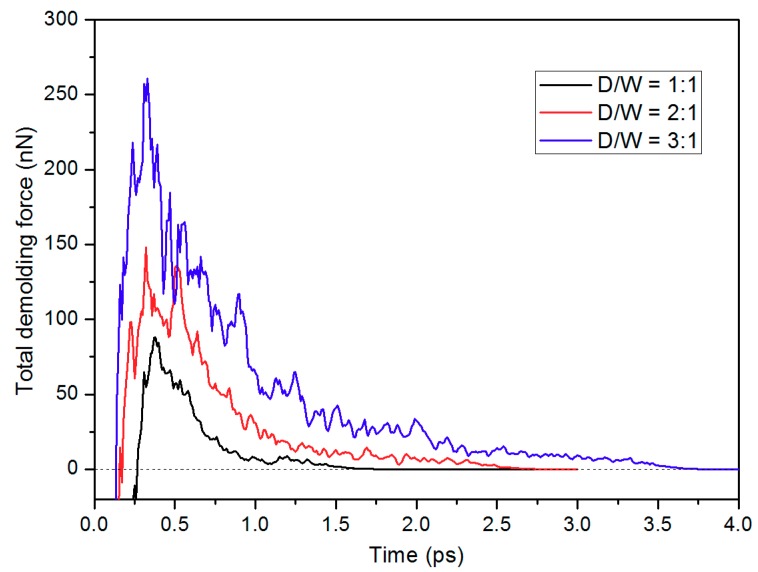
Total demolding forces of PP layer during the separation, with the D/W values of (lower curve) 1:1, (middle curve) 2:1, and (upper curve) 3:1.

**Figure 9:**
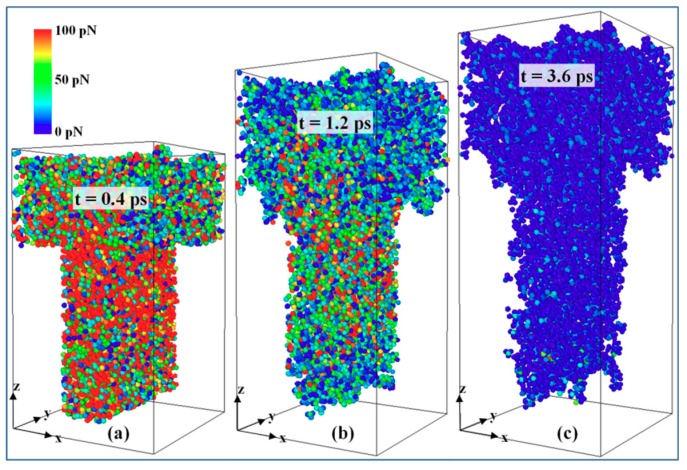
Demolding force per atom in 3:1 nanostructure at a simulation time of (**a**) 0.4 ps, (**b**) 1.2 ps, and (**c**) 3.6 ps.

The total demolding force was investigated by calculating the inner force for each atom. As shown in Figure 8, the demolding force quickly increases to its peak point, prior to the separation of the nanostructure. Afterwards, it gradually decreases to zero shortly when the nanostructure is completely pulled out. Maximum demolding force is observed between the simulation time of 0.3 ps and 0.4 ps. During this period, most of the PP nanostructure is still in the nano-cavity, generating strong adhesion to the surface of both nano-cavity and mold insert. With the increase of D/W, a higher demolding force is generated, since there are more PP molecules surrounding around the interface. Figure 9 shows an example of the density contour for the whole atoms, with the D/W of 3:1 during the demolding process. At 0.4 ps, atoms in the nanostructure show relatively higher demolding force, especially these atoms in the upper area of the nanostructure, due to the strong adhesion. The demolding force per atom decreases at 1.2 ps, while the certain area in nanostructure still shows high demolding force, as shown in Figure 9b. When the nanostructure is pulled out, the demolding force falls to zero correspondingly. 

**Figure 10:**
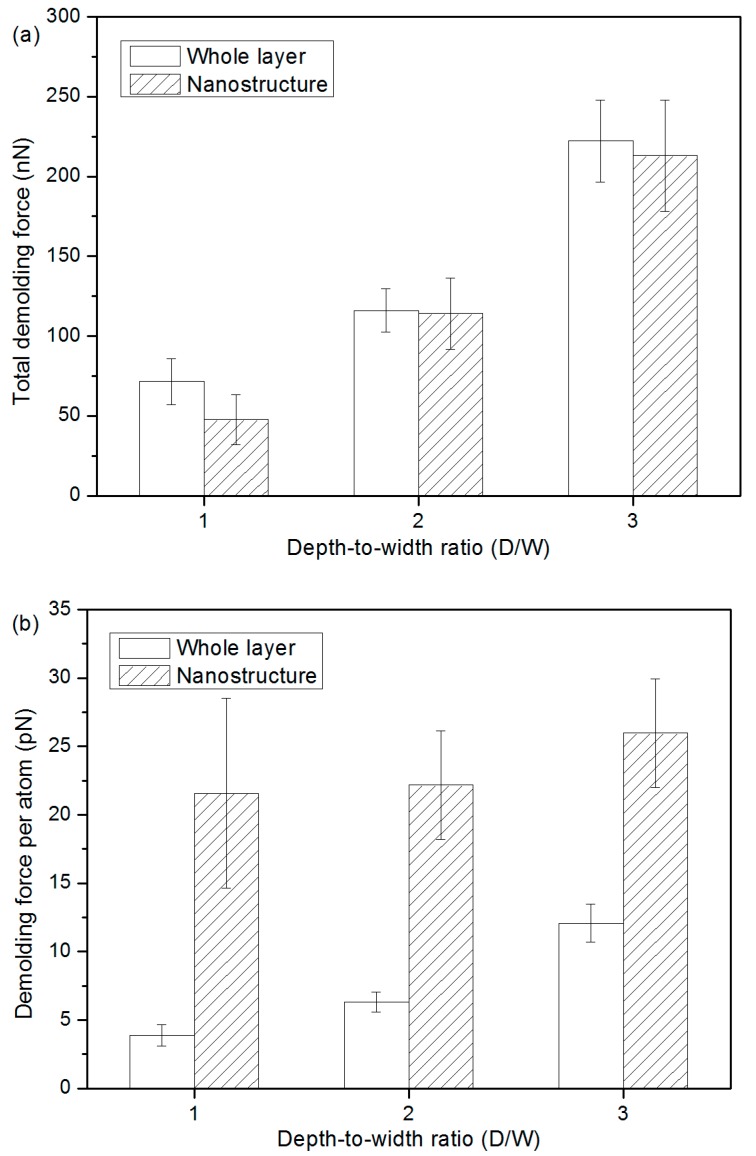
Demolding forces for different D/W, (**a**) the total demolding force and (**b**) the force per atom were calculated as an average value during the simulation time from 0.3 to 0.4 ps, when the highest demolding force occurs.

The total demolding force and the average force were compared during the simulation between 0.3 ps and 0.4 ps in order to better understand the force distribution at the peak point. These forces on each atom of the whole PP layer and the nanostructure were calculated. It is demonstrated in Figure 10a that the total demolding force for the whole PP layer are 71.56 nN, 116.12 nN, and 222.23 nN for 1:1, 2:1, and 3:1 nano-cavity systems, while the total demolding force for PP nanostructure are 47.76 nN, 114.11 nN, and 213.06 nN, respectively. It is demonstrated that the majority of the demolding force comes from the adhesion and friction on the nanostructure, especially when the values of the D/W are 2:1 and 3:1. Although the average force per atom on the nanostructure has a relatively high standard deviation, the demolding force is nevertheless much higher than the average value of the whole layer. With the increases of D/W, total demolding force and average force on each atom both increase. It means that a higher resistance is generated at that time. Similar results on the demolding force distribution can also be observed in other simulation times.

## 4. Conclusions

In this present work, molecular dynamics simulation system that consisted of a PP layer and a nickel mold insert layer was constructed to study the demolding process of the nanostructure in injection molding. The simulation results show that there is hardly any separation of PP molecules in the nano-cavity at the early demolding stage. The separation first happens at the shoulder of the nanostructure, especially for the structure with a high D/W. The nanostructure is stretched since the velocity of PP nanostructure is relatively lower than the whole PP layer. Analyzing the radius of gyration also stretches the PP chains in the whole layer. Moreover, an obvious drop in density is found after the demolding. During the separation, the largest increase in the radius of gyration is approximately 28.3%. The lowest velocity is observed in the 3:1 nanostructure due to the strong interfacial adhesion. Nanostructure deformation gradually occurs, which results in a slight expansion at the shoulder eventually. When the D/W is higher, the adhesion energy that is generated by the non-bonded interaction is higher. It means more demolding time is required for the complete separation. The demolding force increases quickly to its peak point before the separation of the nanostructure. Maximum force can be observed between 0.3 ps and 0.4 ps, with the total force of 71.56 nN, 116.12 nN, and 222.23 nN for 1:1, 2:1, and 3:1 nano-cavity systems. Atoms in nanostructure, especially in the upper area, show relatively higher demolding force. The majority of the force comes from the adhesion and friction on the nanostructure due to the interfacial interaction. With the increases of D/W, total demolding force and demolding force per atom are both higher.
